# Infant and young child feeding practice among mothers of children age 6 to 23 months in Debrelibanos district, North Showa zone, Oromia region, Ethiopia

**DOI:** 10.1371/journal.pone.0257758

**Published:** 2021-09-24

**Authors:** Mathewos Mekonnen, Tadele Kinati, Kumera Bekele, Bikila Tesfa, Dejene Hailu, Kemal Jemal

**Affiliations:** Department of Nursing, College of Health Sciences, Salale University, Fiche, Oromia Region, Ethiopia; Zurich University of applied sciences, School of Health Professions, Leitung Forschunsstelle Gesundheitswissenschaften/Head of health sciences research, SWITZERLAND

## Abstract

**Background:**

Inappropriate infant and young child feeding (IYCF) practice is the leading cause of malnutrition in children. Data is needed to identify children at risk of poor feeding practice and to target interventions to improve IYCF practices. Therefore, this study aimed to assess IYCF practice and associated factors among mothers of children age 6 to 23 months in Debrelibanos district, north Showa zone, Oromia region, Ethiopia.

**Method:**

A community-based cross-sectional study design was conducted among 380 mothers of children age 6 to 23 months from March 1 to April 5, 2019. A simple random sampling technique was used to select the respondents. Data was collected using a structured interviewer-administered questionnaire that had been pretested. The data was entered into Epi-Data 3.1 and then transferred to SPSS 21 for analysis. Descriptive statistical analysis was done, and an association between an outcome variable and independent variables was examined in logistic regression models.

**Result:**

Overall, 65.8% of mothers practiced appropriate IYCF practice. The study revealed that 70.5% of children started breastfeeding within one hour of birth, and 61.6% were breastfed exclusively for six months. Among studied mothers, 79.5% continued to breastfeed their children until 2 years, and 69.2% of the participants started complementary feeding timely at six months. Minimum dietary diversity was observed in 19.2% of children, while minimum meal frequency was found in 79.2%. The majority of mothers (77.6%) fed their babies with bottles. Mother’s educational status of primary school [AOR = 4.50, 95% CI: (1.38,14.61)], husband’s occupation being merchant [AOR = 6.45, 95% CI: (1.51, 27.59)]; antenatal care follows up [AOR = 3.15, % CI: (1.22, 8.12)], radio/television ownership [AOR = 7.41, 95% CI: (2.86, 19.20)], child’s sex being female [AOR = 4.78, 95% CI: (2.26, 10.064) and sufficient knowledge on child feeding [AOR = 2.82, 95% CI: (1.27, 26.26)] were independent predictors for appropriate IYCF practice.

**Conclusion:**

The prevalence of appropriate infant and young child feeding practice indicators was found to be rather high among the mothers in this study. The use of a bottle to feed babies, in particular is very common among the mothers who were studied. To address child malnutrition, it is critical to educate families about proper IYCF practices. This study suggests that mothers be properly educated about IYCF recommendations at health care facilities during their visits, as well as the promotion of appropriate IYCF through various media.

## Introduction

Malnutrition accounts for around two-thirds of all deaths among children under the age of five worldwide. Each year, more than 10 million children die as a result of malnutrition; 98% of these deaths occur in underdeveloped countries [1]. Although the risk of death is higher in stunted children, it is exacerbated when wasting and stunting occur at the same time [2]. Children’s health, physical growth, cognitive development, and school performance are all compromised by chronic malnutrition [3]. Stunting in childhood has also been linked to a higher risk of death for mothers in the future, as well as poor pregnancy outcomes such as preterm and low birth weight child [4, 5]. Furthermore, it has a detrimental impact on adults’ economic productivity as well as the country’s overall gross domestic product [6]. In Ethiopia, according to 2016 Ethiopian Demographic Health Survey (EDHS) 38 percent and ten percent of children under the age of five are stunted and wasted, respectively [7]. Undernutrition is rampant in Ethiopia, owing primarily to inappropriate feeding practice [8].

World Health Organization (WHO) and the United Nations Children’s Education Fund (UNICEF) have demonstrated that basic nutrition programs can avert a quarter of child mortalities and one-third of chronic malnutrition. As a result, UNICEF and WHO have established a global strategy for optimal IYCF to combat child malnutrition and mortality [9]. Early initiation of breastfeeding; exclusive breastfeeding during the first 6 months of life; timely introduction of complementary feeding at six months; continued breastfeeding after complementary foods is introduced; adequate dietary diversity in complementary foods; adequate frequency of foods and nonuse of bottle for feeding baby are important aspects of IYCF in the first 2 years of life [10–12].

According to the 2016 EDHS, 58 percent of children were exclusively breastfed during the first six months of their lives. At two years, 76 percent of mothers continued to breastfeed their infants, while 60 percent of mothers began supplemental feeding on time. Furthermore, 14 percent and 45 percent of mothers, respectively, had a minimum dietary and meal frequency [7].

Efforts to enhance the nutritional status of children in Ethiopia have been made at various times, including the adoption of IYCF recommendations [13]. However, these attempts have been ineffective in bringing about significant and long-term changes in IYCF practices. In a study conducted in Shashamane, Ethiopia, for instance, the prevalence of inappropriate IYCF practice was found to be 67.9% [14].

To build programs that could direct and impact public health initiatives, combined data on key elements of IYCF practice is required. Previous studies on child feeding, on the other hand, mainly focused on child feeding components separately. There are very few studies on the IYCF index’s combined variables based on WHO-identified key indicators in Ethiopia. As a result, this study aimed to assess IYCF practice and associated factors among mothers of children age 6 to 23 months in Ethiopia’s Debrelibanos district, North Showa zone, Oromia region.

## Methods

### Study design, period, and area

From March 1 to April 5, 2019, a community-based cross-sectional study was undertaken. The study was conducted in Ethiopia’s Oromia region, in the Debrelibanos district of the North Showa zone. The district is 90 kilometers from Ethiopia’s capital, Addis Ababa. One urban and ten rural kebeles make up the district. The population of the Debrelibanos district was estimated to be 64,305, according to unpublished figures acquired from the district. The ratio of males to females was 1:1. The reproductive age group (15–45 years) accounts for 22.23 percent of the female population, with 3,672 women having children under the age of two years.

### Study population

The study population for this study was all mothers with children age 6 to 23 months who had lived in the district for more than six months. Mothers who were critically ill and unable to communicate were excluded from the study.

### Sample size and sampling technique

The sample size for this study was calculated using a formula for a single population proportion, which took into account the power of 80%, the confidence interval level of 95%, the margin of error of 5%, and the prevalence of appropriate IYCF practice 32.1% [14]. The sample size was 385 after adjusting for the non-response rate. Due to the small number of *kebeles* in the district, all of them were considered. The sample size was then proportionally allocated to the size of each population size of *kebele*. Finally, the participants were selected using simple random sampling.

### Data collection tools and procedure

The instrument was developed using previous literature, national guidelines, and the WHO’s IYCF practice guideline, and it was pretested before actual data collection. Mother and child demographic characteristics, household characteristics, maternal and child health care service use, paternal involvement in child feeding, knowledge of IYCF, an attitude of mothers toward IYCF, and IYCF practices were all included in the questionnaire. To assess IYCF practice, seven WHO indicators were used. For each indicator, the appropriate feeding practice was assessed based on compliance with WHO recommended practices. A 24-hour recall approach was used to assess minimum dietary diversity, minimum meal frequency, continued breastfeeding, and bottle feeding. However, a child’s lifetime historical recall approach was used to elicit early initiation of breastfeeding, exclusive breastfeeding, and timely initiation of complementary feeding. Under the supervision of two BSc nurses, face-to-face interviews by eight female diploma nurses were conducted. The data collectors and supervisors received training on the study’s purpose and data collection methodology.

### Operational definitions

**Early initiation of breastfeeding**: initiation of breast milk within one hour after delivery

**Exclusive breastfeeding**: receiving only breast milk, except drops or syrup consisting of vitamins, minerals, or medicines up to six months.

**Timely initiation of complementary feeding**: introducing solid or semi-solid food at 6–8 months

**Minimum dietary diversity**: the proportion of children 6–23 months of age who consume four or more out of seven standard groups. Food groups include grains, roots, and tubers; legumes and nuts; dairy products; flesh foods, eggs; vitamin A-rich fruits and vegetables; other fruits and vegetables [15].

**Minimum meal frequency**: children age 6–23 months who receive solid, semi-solid, or soft foods the minimum number (6–8 months, 2 times; 9–23 months, 3 times for breastfed children and 6–23 months, 4 times for non-breastfed children) [15].

**Continued breastfeeding**: Proportion of children 6–23 months of age who received breast milk

**Bottle feeding:** Proportion of children 6–23 months of age who are fed with a bottle.

**Appropriate IYCF practice:** was measured from seven WHO indicators (i.e., early initiation of breastfeeding, exclusive breastfeeding, no bottle feeding, minimum meal frequency, minimum dietary diversity, timely introduction of complementary foods, and continued breastfeeding in children 6–23 months). If a child fulfilled at least mean or four of the seven criteria, it was classified as appropriate IYCF practice [11, 14].

**Knowledge about IYCF**: was measured from seven knowledge questions about child feeding. Based on the summation score, a score above 50% was considered as having sufficient knowledge about IYCF

### Data processing and analysis

Completeness and consistency of responses were checked on filled questionnaires. The data was manually pre-coded, then entered into Epi-Data version 3.1 statistical software, which was then transferred to SPSS 21 for analysis. Data was edited by checking for missing values and outliers after running frequencies.

During analysis the age-appropriate IYCF indicator questions were recoded into the same variables and coded as (yes = 1 and no = 0) to make the variable dichotomous, then the coded response was computed to get the mean response of seven IYCF indicator questions, after that the mean response of the seven IYCF questions was computed to get the overall mean response of seven IYCF practice, then those who responded above the mean were coded as (1 appropriate IYCF practice and 0 for inappropriate IYCF practice).

To measure knowledge on IYCF; seven knowledge questions recoded as (yes = 1 and no = 0) were used to construct a composite score. Based on the summation score, a score above 50% was considered as having sufficient knowledge about IYCF

The results of a descriptive statistical analysis were given in percentages, means, standard deviations, using tables. Binary logistic regression was used to perform bivariate analysis between dependent and independent variables. The P-value of ≤ 0.25 was taken as a cut-off point to select eligible variables for the multiple logistic regression analysis to control for potential confounders. Association was expressed as crude odds ratios (COR) and adjusted odds ratio (AOR), and their corresponding 95% confidence intervals (CIs) were obtained from logistic regression models. Odds ratios (OR) were reported together with their 95% CI, and statistical significance was deemed at P-value < 0.05.

### Ethical considerations

Salale University Ethical Review Committee provided ethical approval for this study. The district issued a permission letter. Mothers gave their written/ fingerprint consent. The participants were told that they had the freedom to participate or not. Participants were encouraged to be as honest as possible because their information is valuable to the district, region, and country as a whole. By omitting their names from the questionnaire, the participants’ confidentiality was preserved

## Result

### Maternal socio-demographic characters

A total of 380 mothers with children age between 6 to 23 months participated in the study with a response rate of 98.7%. The mean age of the mothers was 28.36 (±SD = 5.78) years. The majority of the participants 308 (81.1%) were housewives /farmers, with an average monthly income of nearly 1,000 Ethiopian Birr (ETB). The Orthodox made up 339 (89.2%), while the Protestants made up 36 (9.5%). About a third of the respondents (34.7%) couldn’t read or write (Table 1).

**Table 1:** Socio-demographic characteristics of the study participants at Debrelibanos district, north Showa, Oromia, and Ethiopia.

Variables		Frequency	Percent
Age of mother	15–24	88	23.2
	25–34	216	56.8
	35–45	76	20.0
Marital Status	Married	346	91.0
	Divorced	22	5.8
	Other ^a^	12	3.2
Religion	Orthodox	339	89.2
	Protestant	36	9.5
	Others ^b^	5	1.3
Mother’s educational status	Unable to read & write	132	34.7
	Able to read &write	25	6.6
	Primary school	87	22.9
	Secondary school	97	25.5
	College and above	39	10.3
Mother’s occupation	Housewife/farmer	308	81.1
	Government employee	35	9.2
	Merchant	30	7.9
	Others ^c^	7	1.8
Husband’s education status	Unable to read & write	122	32.1
	Able to read &write	14	3.7
	Primary school	80	21.0
	Secondary school	93	24.5
	College and above	71	18.7
Husband’s occupation	Farmer	253	66.6
	Merchant	54	14.2
	Government employee	51	13.4
	Others ^d^	22	5.8
Family income in ETB	< = 1000	183	48.2
	1001–2000	74	19.5
	2001–3000	40	10.5
	> = 3001	83	21.8
Radio/TV ownership	No	123	32.4
	Yes	257	67.6

^a^ single and widowed

^b^ Muslim and wakefata

^c^ private employee and daily laborer

^d^ private employee and daily laborer; ETB, Ethiopian Birr.

### Maternal, child, and household characteristics

Three-quarters of the mothers in the study, 287 (75.5%), were multipara, and approximately half of the families, 203 (53.4%), had one or two children. Males account for half (50.8%) of the children who participated in the study. The majority of the children in the study, 267 (64.5%), were between the ages of 12 and 23 months. The mean of children was 13.43 months (SD = 5.216) at the time of the study. A significant number of husbands, 149 (39.2%), were not involved in child feeding. More than three-quarters of the mothers 263, (69.2%) had sufficient knowledge on IYCF practice. Two hundred thirty-four (61.6%) of mothers had a positive attitude towards IYCF practice (Table 2).

**Table 2:** Maternal, child, and household characteristics of the study participants, Debrelibanos district, north Showa, Oromia, Ethiopia.

Variables		Frequency	Percent
Parity	Primpara	93	24.5
	Multipara	287	75.5
ANC follow up	No	69	18.2
	Yes	311	81.8
Place of delivery	Home	111	29.2
	Health facility	269	70.8
Current pregnancy status	No	338	88.9
	Yes	42	11.1
Sex of child	Male	193	50.8
	Female	187	49.2
Age of child	6–8	81	21.3
	9–12	57	15.0
	12–23	242	63.7
Number of children in the family	1–2	203	53.4
	3–4	117	30.8
	> = 5	60	15.8
Paternal involvement in child feeding	No	149	39.2
	Yes	231	60.8
Mother’s knowledge on IYCF	Insufficient	117	30.8
	Sufficient	263	69.2
Mother’s attitude towards IYCF	Negative	146	38.4
	Positive	234	61.6

ANC, Antenatal care; IYCF, infant and young child feeding.

### Infant and young child feeding practices

Nearly two-thirds of mothers (65.8%) practiced appropriate IYCF. In terms of IYCF components, the majority of mothers, 268 (70.5%), reported starting breastfeeding within one hour of birth, and 234 (61.6%) gave their child solely breast milk for the first six months. A considerable number of mothers 295 (77.6%) fed their babies with a bottle. At six months, over two-thirds of the mothers 263(69.2%) started complementary feeding timely. Only 73 (19.2%) of the respondents met the minimum dietary diversity requirements. The majority of mothers, 302 (79.5%), continued breastfeeding, and the minimum meal frequency was 301 (79.2%) for complementary food (Table 3).

**Table 3:** Infant and young child feeding practice components among mothers with children age between 6 to 23 months at Debrelibanos district, north Showa, Oromia, Ethiopia.

IYCF Component		Frequency	Percent
Early initiation of breastfeeding	Yes	268	70.5
	No	112	29.5
Exclusive breastfeeding	Yes	234	61.6
	No	146	38.5
No bottle-feeding	Yes	85	22.4
	No	295	77.6
Timely initiation of complementary food	Yes	263	69.2
	No	117	30.8
Minimum dietary diversity	Yes	73	19.2
	No	307	80.8
Minimum meal frequency	Yes	301	79.2
	No	79	20.8
Continued breastfeeding	Yes	302	79.5
	No	71	20.5
IYCF practice	Appropriate	250	65.8
	Inappropriate	130	34.2

IYCF, infant and young child feeding.

### Factor associated with infant and young infant feeding practices

In binary logistic regression model, age of mother, educational status of the mother, occupation of mother, husband’s educational status, husband’s occupation, family radio/television ownership, mother’s parity, ANC follow up, place of delivery, sex of the child, paternal involvement in child feeding, number of children in the household, knowledge on IYCF and attitude towards IYCF practices were significantly associated with IYCF practices (Table 4).

**Table 4:** Binary and multiple variables logistic regression analysis of factors associated with IYCF practices among mothers with children age between 6 to 23 months at Debrelibanos district, north Showa, Oromia, Ethiopia.

Variables	Category	Appropriate IYCF Practice		COR (95%CI)	AOR (95%CI)
		No (N)	Yes (N)		
Age of mother	15–24	23	65	1	
	25–34	70	146	0.74 (0.42, 1.29)	
	35–45	37	39	0.37 (0.19, 0.72)	
Mother’s education	Unable to read & write	82	50	1	1
	Able to read &write	3	22	12.03 (3.42, 42.25)	1.31 (.232, 7.36)
	Primary School	10	77	12.63 (5.98, 26.65)	4.50 (1.38, 14.61) *
	Secondary School	25	72	4.72 (2.66, 8.40)	1.12 (0.31, 4.10)
	College and above	10	29	4.76 (2.14, 10.59)	0.58 (0.12, 2.81)
Mother’s occupation	Housewife/Farmer	118	190	1	
	Government employee	7	28	2.48 (1.05, 5.87)	
	Merchant	3	27	5.59 (1.66, 18.83)	
	Others ^a^	2	5	1.55 (0.30, 8.13)	
Husband’s education	Unable to read & write	82	40	1	
	Able to read &write	2	12	12.30 (2.62, 57.60)	
	Primary school	11	69	12.86 (6.14, 26.96)	
	Secondary school	16	77	9.87 (5.11, 19.05)	
	College and above	19	52	5.61 (2.94, 10.72)	
Husband’s occupation	Farmer	110	143	1	1
	Merchant	6	48	6.15 (2.54, 14.90)	6.45 (1.51, 27.59) *
	Government employee	12	39	2.50 (1.25, 5.00)	0.99 (0.35, 2.81)
	Others ^a^	2	20	7.69 (1.76, 33.61)	
Radio/TV ownership	No	89	34	1	1
	Yes	41	216	13.79 (8.22, 23.13)	7.41 (2.86, 19.20) *
Parity	Primary	17	76	1	
	Multipara	113	174	0.34 (0.19, 0.61)	
ANC follow up	No	44	25	1	1
	Yes	86	225	4.60 (2.66, 7.98)	3.15 (1.22, 8.12) *
Place of delivery	Home	61	50	1	
	Health facility	69	200	3.54 (2.23, 5.62)	
Current pregnancy status	No	119	219	1	
	Yes	11	31	1.53 (0.74, 3.16)	
Sex of child	Male	83	110	1	1
	Female	47	140	2.25 (1.45, 3.48)	4.78 (2.26,10.06) *
Age of child	6–8	26	55	1	
	9–12	26	31	0.56 (0.28, 1.13)	
	12–23	78	164	0.99 (0.58, 1.70)	
Number of children in the family	1–2	53	150	1	
	3–4	54	63	0.41 (0.26, 0.67)	
	> = 5	23	37	0.57 (0.31, 1.04)	
Paternal involvement	No	76	73	1	
	Yes	54	177	3.41 (2.19, 5.31)	
Knowledge on IYCF	Insufficient	72	45	1	1
	Sufficient	58	205	5.66 (3.52, 9.08)	2.82 (1.27, 6.26) *
Attitude towards IYCF	Negative	69	77	1	
	Positive	61	173	2.54 (1.64,3.93)	

ANC, Antenatal care; IYCF, infant and young child feeding; TV, Television; COR; Crude odds ratio; AOR, Adjusted odds ratio

*Shows significant association for multivariate logistic regressions at 95% CI

In multiple logistic regression, mothers with primary school educational status were more likely to practice appropriate IYCF practices than odds of illiterate mothers [AOR = 4.50, 95% CI: (1.38, 14.61)]. A mother whose husband was a merchant was more likely to practice appropriate feeding than a mother whose husband was a farmer [AOR = 6.47, 95% CI: (1.51, 27.59)]. Mothers from the family who had radio/television at home were seven-time more likely to practice proper feeding practices [AOR = 7.41, 95% CI: (2.86, 19.20)]. Mothers who made ANC follow up during pregnancy were three times more likely to practice appropriate IYCF practices [AOR = 3.15, 95% CI: (1.22, 8.12)]. Mothers who had sufficient knowledge of IYCF were more likely to practice appropriate feeding practices [AOR = 2.82, 95% CI: (1.27, 26.26)]. Finally, having a female child was found to be a predictor for appropriate IYCF practices [AOR = 4.78, 95% CI: (2.26, 10.06)] (Table 4)

## Discussion

In order to reduce malnutrition in a developing country like Ethiopia, safe, adequate and acceptable infant and child feeding practice is vital. For this reason, WHO and UNICEF have recommended core infant feeding practices. This study assessed IYCF practice and associated factors among mothers of children age 6 to 23 months using seven WHO indicators. In the current study, a significant number of mothers practiced appropriate IYCF practice. Furthermore, although, the practice of bottle feeding is extremely high, the practice of recommended feeding practices is relatively better as compared to the national prevalence and other previous studies’ report in majority of IYCF practice components. Mother’s educational status of being primary school, husband’s occupation being merchant, ANC follow up, possession of radio/television, child’s sex being female and sufficient knowledge on child feeding were independent positive predictors of appropriate IYCF practice.

The current study’s prevalence of appropriate IYCF practice (65.8%) is higher than a study conducted in Ethiopia’s Shashamane Woreda, where 32.1% of caregivers practiced appropriate IYCF practice [14]. The inconsistency could be due to the length of time between studies, and in Ethiopia, the number of mothers seeking maternal health care has increased considerably as a result of the country’s ongoing promotion of free maternal services, providing a favorable chance for health professionals to promote IYCF practice. Even though the WHO guideline on IYCF practice [16] does not specify minimum standards to be met or the percentage that should be considered for public health importance, it is understandable that all children (6–23 months) should follow all of the recommended feeding practices.

Early initiation of breastfeeding is important for both the mother and the child. As a result, it is suggested that children be put to the breast as soon as possible after birth, preferably within one hour of birth. In this study, a little more than two-thirds of mothers (70.5%) started breastfeeding within one hour of delivery. The finding is nearly identical to the national prevalence (73%) according to the 2016 EDHS [7]. The result is, however, slightly lower than that of a study conducted in Mekelle town, Ethiopia (78%) [17]. This could be due to the fact that the previous study was conducted in a town where healthcare and the media are more readily available than in the current study area, where the majority of the study population comes from rural areas.

The prevalence of exclusive breastfeeding was 61.6% in this study. This finding is comparable to what was found at the national level (58%) in the EDHS 2016 and Mekelle (60.8%) [7, 17]. However, it is lower than another study conducted in Ethiopia (87.8%) [14]. The discrepancy could be attributed to differences in exclusive breastfeeding measurement, where the former study assessed exclusive breastfeeding based on 24-hour recall, and the current study assessed retrospectively asking about exclusive breastfeeding.

The introduction of nutritionally adequate and safe complementary foods to infants and young children enhances growth and development. According to the finding, 69.2% of the children studied received complementary foods timely. This finding is in line with reports from developing countries such as Nepal (70%) [18] and Bangladesh (71%) [19]. However, the finding is lower when compared to other African countries, including Uganda (75%), Tanzania (92.3%), and Kenya (81%) [20–22] but higher than other studies conducted in Ethiopia (51–62.8%) [23–25], India (55.1%) [26] and national prevalence as per EDHS, 2016 (60%) [7]. Disparity across the studies in reporting time of initiating complementary food could be due to the use of different methods of measurement time of initiation: at the sixth-month or using reference period 6–8 months.

According to WHO’s IYCF recommendation, breastfeeding should be continued for the first two years, along with complementary food. The current study found that 79.5% of the mothers in the study continued to breastfeed their children. This finding is consistent with the national prevalence in Ethiopia (76%) [7]. It is, however, higher than the 43.3% reported in an Indian study [26]. This could be due to the Ethiopian context’s intensified health education package provided by health extension workers.

Dietary diversity is a proxy for adequate micronutrient density of foods. Food intake from at least four of the seven food groups is assessed in children aged 6 to 23 months as minimum dietary diversity. This study’s minimum food diversity (19.2%) was greater than the national prevalence (14%) [7] and a study in the Gorche district of southern Ethiopia (10.6%) [27]. However, it was lower than the findings of studies conducted in Bangladesh (41.5%) and Sri Lanka (71.1%) [19, 28]. This could be due to a lack of availability to the required food combination.

Minimum meal frequency examines the number of times children received foods other than breast milk. The minimum number is specific to the age and breastfeeding status of the child. Accordingly, the minimum meal frequency in this study (79.2%) is almost similar to India (78%) [26] and Bangladesh (81.1%) [19]. However, it is lower than Sri Lanka (88.3%) [28] and Ethiopia (45%) according to 2016 EDHS [7].

Using a bottle with a nipple to feed an infant, a practice that is discouraged increases the child’s risk of illness and decreases the child’s desire to breastfeed, perhaps resulting in a decrease in milk output. The majority of mothers (77.6%) used bottles to feed their babies, according to the findings of the study. This prevalence is higher than that reported by the EDHS in 2016 (14%) (7), Western Uganda (10%) [20], and a study conducted in rural Ethiopia (39.8%) [29]. The highest finding could be attributed to the area’s highest milk production, and cow’s milk is more convenient to provide to children using bottles than other foods.

There was an association between ANC follow-up and appropriate IYCF practices among mothers in this study. This result is supported by research from Ethiopia [14] and Nepal [30]. The association may be due to the fact that mothers who had ANC visits may have received related health information from their health care providers. As a result, ensuring that pregnant mothers receive recommended ANC could be a useful strategy for improving IYCF practice.

In the current study, having a radio or television had a significant relationship with appropriate IYCF practice. The result is supported by secondary data analysis from the EDHS and another study from Northwest Ethiopia [31, 32]. Media ownership was also identified as a key driver of IYCF practice in an Indian study [33]. Because the media is generally regarded as a trustworthy source of health and nutrition information and such messages are more likely to be implemented. Mothers who have radio are also more likely to be exposed to IYCF education provided through mass media.

Appropriate infant and young child feeding practice was found to be significantly associated with having a female child in the current study, with females four times more likely to be appropriately fed than their male counterparts. This result is consistent with a study conducted in the Ethiopian town of Asella and India, which found that being female, was positively related to receiving the proper IYCF practice [34]. Some authors [35] speculated that women may believe that boy infants have greater nutritional needs and hence require complementary feeding at an earlier age, resulting in an untimely introduction of complementary food and therefore affecting exclusive breastfeeding practice. Male children are weaned earlier than female children, according to another Algerian study [36]. As a result, mothers must be educated that breast milk alone is adequate to provide essential nutrition for infants’ optimal growth and development during the first six months of life, and that breastfeeding should be continued until the age of two years, irrespective of sex.

The mother’s educational status was associated with appropriate IYCF practice. This result is consistent with findings from prior studies in Nepal, Pakistan, and Ethiopia [37–39]. This could be because educated women grasp nutrition education better than less educated or uneducated mothers. Furthermore, educated mothers are more likely than their counterparts to read books, pamphlets, and periodicals, as well as be exposed to nutrition education concerning IYCF via mass media. Hence mothers with a lower level of education should be given extra support to help maintain to practice appropriate IYCF practice.

Being a merchant is positively associated with appropriate IYCF practice when it comes to the husband’s occupation. This could be related to the fact that merchants in the study area have a relatively high income, which could lead to increased purchasing power and easier access to a variety of foods as they begin complementary food, as opposed to other occupations.

Appropriate IYCF practice was associated with having sufficient knowledge on IYCF practice. Another study in rural Ethiopia [40] came up with similar results. The likely reason could be linked to the importance of knowledge in empowering mothers to resist external pressures and interferences from traditional views and misconceptions about IYCF. The study finding could support reinforcing nutrition education through tailoring with behavioral change and communication components to address the community misunderstanding towards IYCF practice. As a result, efforts to improve mothers’ IYCF knowledge should be escalated.

There are some limitations to this study: The study’s cross-sectional design, for example, makes it difficult to assess the cause-and-effect link between possible causes and the outcome. Because of recall and social desirability biases, the 24-hour recall approach may result in an overestimation of the proportion of IYCF practices. Discrepancies in the measurement of feeding practices, as well as the real meaning of each practice, also make building and interpreting composite feeding practices challenging. Another limitation of the study is its generalizability; the population was sampled from only one Woreda, thus it may not be representative of the region or country.

## Conclusion

In conclusion, the prevalence of appropriate infant and young child feeding practices indicators was found to be rather high among the mothers in this study. Bottle feeding in particular is, very common among the women who were studied. Mother’s educational status, husband’s merchant occupation, family radio/television ownership, ANC follow-up, child’s sex, and sufficient knowledge of IYCF were all independent predictors of appropriate IYCF practice. In order to combat child malnutrition, it is critical to educate mothers about appropriate IYCF practices. This study suggests that health workers educate mothers about IYCF guidelines during antenatal care, particularly for mothers with lower educational levels; it also recommends that appropriate IYCF be promoted through mass media.

## Supporting information

S1 Data Data used in the study. (SAV)

S1 File Questionnaire. (DOCX)

## References

[1] UNICEF. The state of the world’s children 2007: women and children: the double dividend of gender equality: UNICEF; 2006.

[2] McDonald CM, Olofin I, Flaxman S, Fawzi WW, Spiegelman D, Caulfield LE, et al. The effect of multiple anthropometric deficits on child mortality: a meta-analysis of individual data in 10 prospective studies from developing countries. The American journal of clinical nutrition. 2013;97(4):896–901. doi: 10.3945/ajcn.112.047639 23426036

[3] Opoola F, Adebisi SS, Ibegbu AO. The Study of nutritional status and academic performance of primary school children in zaria, Kaduna state, Nigeria. Annal Bioanthropol. 2016;4(2):96

[4] Black RE, Allen LH, Bhutta ZA, Caulfield LE, De Onis M, Ezzati M, et al. Maternal and child undernutrition: global and regional exposures and health consequences. The lancet. 2008;371(9608):243–60. doi: 10.1016/S0140-6736(07)61690-0 18207566

[5] The World Bank. Repositioning Nutrition as Central to Development. A Strategy for Large‐Scale Action. The International Bank for Reconstruction and Development. 2006.

[6] Grantham-McGregor S, Cheung YB, Cueto S, Glewwe P, Richter L, Strupp B, et al. Developmental potential in the first 5 years for children in developing countries. The lancet. 2007;369(9555):60–70. doi: 10.1016/S0140-6736(07)60032-4 17208643 PMC2270351

[7] Central Statistical Agency [Ethiopia], ICF International. Ethiopia Demographic and Health Survey 2016. Addis Ababa, Ethiopia and Calverton, Maryland, USA: Central Statistical Agency and ICF International; 2017.

[8] Benson T, Bellete S, Chanyalew D, Belachew T. An assessment of the causes of malnutrition in Ethiopia. International Food Policy Research Institute Washington DC. 2005 Nov:1–213. 9.

[9] WHO, UNICEF. Global strategy for infant and young child feeding. Geneva: World health organization. 2003.

[10] Chandwani H, Prajapati A, Rana B, Sonaliya K. Assessment of infant and young child feeding practices with special emphasis on IYCF indicators in a field practice area of Rural Health Training Centre at Dabhoda, Gujarat, India. Int J Med Sci Public Health. 2015;4(10):1414–9.

[11] WHO. Indicators for assessing infant and young child feeding practices part 3: country profiles. 2010.

[12] UNICEF. Programming guide, infant and young child feeding, nutrition section, programmes, UNICEF. New York. 2012.

[13] Federal Ministry of Health. National strategy for infant and young child feeding, Ethiopia: Federal Ministry of Health, Family Health Department. 2004

[14] Yonas F. Infant and young child feeding practice status and associated factors among mothers of under 24-month-old children in Shashemene Woreda, Oromia region, Ethiopia. Open Access Library Journal. 2015;2(07):1.

[15] UNICEF. WHO: Indicators for assessing infant and young child feeding practices. Geneva: WHO and UNICEF. 2007.

[16] WHO. Implementing the Global Strategy for Infant and Young Child Feeding: Geneva, 3–5 February 2003: meeting report: World Health Organization; 2003.

[17] Berhe H, Mekonnen B, Bayray A, Berhe H. Determinants of breastfeeding practices among mothers attending public health facilities, Mekelle, northern Ethiopia; a cross-sectional study. International Journal of Pharmaceutical Sciences and Research. 2013;4(2):650.

[18] Joshi N, Agho KE, Dibley MJ, Senarath U, Tiwari K. Determinants of inappropriate complementary feeding practices in young children in Nepal: secondary data analysis of Demographic and Health Survey 2006. Maternal & child nutrition. 2012;8:45–59. doi: 10.1111/j.1740-8709.2011.00384.x 22168518 PMC6860874

[19] Kabir I, Khanam M, Agho KE, Mihrshahi S, Dibley MJ, Roy SK. Determinants of inappropriate complementary feeding practices in infant and young children in Bangladesh: secondary data analysis of Demographic Health Survey 2007. Maternal & child nutrition. 2012;8:11–27. doi: 10.1111/j.1740-8709.2011.00379.x 22168516 PMC6860519

[20] Wamani H, Åstrøm AN, Peterson S, Tylleskär T, Tumwine JK. Infant and young child feeding in western Uganda: knowledge, practices and socio-economic correlates. Journal of tropical pediatrics. 2005;51(6):356–61. doi: 10.1093/tropej/fmi048 15947011

[21] Vitta BS, Benjamin M, Pries AM, Champeny M, Zehner E, Huffman SL. Infant and young child feeding practices among children under 2 years of age and maternal exposure to infant and young child feeding messages and promotions in Dar es Salaam, Tanzania. Maternal & child nutrition. 2016;12:77–90. 27061958 10.1111/mcn.12292 PMC5071773

[22] Kimani-Murage EW, Madise NJ, Fotso J-C, Kyobutungi C, Mutua MK, Gitau TM, et al. Patterns and determinants of breastfeeding and complementary feeding practices in urban informal settlements, Nairobi Kenya. BMC public health. 2011;11(1):1–11. doi: 10.1186/1471-2458-11-396 21615957 PMC3118248

[23] Shumey A, Demissie M, Berhane Y. Timely initiation of complementary feeding and associated factors among children aged 6 to 12 months in Northern Ethiopia: an institution-based cross-sectional study. BMC public health. 2013;13(1):1–7. doi: 10.1186/1471-2458-13-1050 24195592 PMC4228254

[24] Yemane S, Awoke T, Gebreslassie M. Timely initiation of complementary feeding practice and associated factors among mothers of children aged from 6 to 24 months in Axum town, north Ethiopia. Int J Nutr Food Sci. 2014;3(5):438–42.

[25] Semahegn A, Tesfaye G, Bogale A. Complementary feeding practice of mothers and associated factors in Hiwot Fana Specialized Hospital, Eastern Ethiopia. The Pan African Medical Journal. 2014;18. 10.11604/pamj.2014.18.143.3496 PMC4236776 25419281

[26] Sinhababu A, Mukhopadhyay DK, Panja TK, Saren AB, Mandal NK, Biswas AB. Infant-and young child-feeding practices in Bankura district, West Bengal, India. Journal of health, population, and nutrition. 2010;28(3):294. doi: 10.3329/jhpn.v28i3.5559 20635641 PMC2980895

[27] Dangura D, Gebremedhin S. Dietary diversity and associated factors among children 6–23 months of age in Gorche district, Southern Ethiopia: Cross-sectional study. BMC pediatrics. 2017;17(1):1–7. doi: 10.1186/s12887-016-0759-7 28068965 PMC5223415

[28] Senarath U, Agho KE, Akram DeS, Godakandage SS, Hazir T, Jayawickrama H, et al. Comparisons of complementary feeding indicators and associated factors in children aged 6–23 months across five South Asian countries. Maternal & child nutrition. 2012;8:89–106. doi: 10.1111/j.1740-8709.2011.00370.x 22168521 PMC6860856

[29] Roba KT, O’Connor TP, Belachew T, O’Brien NM. Infant and young child feeding (IYCF) practices among mothers of children aged 6–23 months in two agro-ecological zones of rural Ethiopia. Int J Nutr Food Sci. 2016;5(3):185–94.

[30] Khanal V, Sauer K, Zhao Y. Determinants of complementary feeding practices among Nepalese children aged 6–23 months: findings from demographic and health survey 2011. BMC pediatrics. 2013;13(1):1–13. doi: 10.1186/1471-2431-13-131 23981670 PMC3766108

[31] Beyene M, Worku AG, Wassie MM. Dietary diversity, meal frequency and associated factors among infant and young children in Northwest Ethiopia: a cross-sectional study. BMC public health. 2015;15(1):1–9. doi: 10.1186/s12889-015-2333-x 26433689 PMC4592571

[32] Aemro M, Mesele M, Birhanu Z, Atenafu A. Dietary diversity and meal frequency practices among infant and young children aged 6–23 months in Ethiopia: a secondary analysis of Ethiopian demographic and health survey 2011. Journal of nutrition and metabolism. 2013. doi: 10.1155/2013/782931 24455218 PMC3878383

[33] Malhotra N. Inadequate feeding of infant and young children in India: lack of nutritional information or food affordability? Public health nutrition. 2013;16(10):1723–31. doi: 10.1017/S1368980012004065 22939461 PMC10271245

[34] Sasie S, Oljira L, Demena M. Infant, and young child feeding practice and associated factors among mothers/caretakers of children aged 0–23 months in Asella Town, South East Ethiopia. Journal of Family Medicine. 2017;4(5):11–22.

[35] Venancio SI, Monteiro CA. Individual and contextual determinants of exclusive breastfeeding in São Paulo, Brazil: a multilevel analysis. Public Health Nutrition. 2006;9(1):40–6. doi: 10.1079/phn2005760 16480532

[36] Aakre I, Lilleengen AM, Aarsand ML, Strand TA, Barikmo I, Henjum S. Infant feeding practices in the Saharawi refugee camps Algeria, a cross-sectional study among children from birth to six months of age. International breastfeeding journal. 2016;12(1):1–10. doi: 10.1186/s13006-016-0098-1 28149322 PMC5273854

[37] Chapagain R. Factors affecting complementary feeding practices of Nepali mothers for 6 months to 24 months children. Journal of Nepal Health Research Council. 2013. 24362612

[38] Hasnain S, Majrooh MA, Anjum R. Knowledge and practices of mothers for complementary feeding in babies visiting pediatrics outpatient department of Jinnah hospital, lahore. Biomedica. 2013;29(4).

[39] Tessema M, Belachew T, Ersino G. Feeding patterns and stunting during early childhood in rural communities of Sidama, South Ethiopia. Pan African Medical Journal. 2013;14(1). doi: 10.11604/pamj.2013.14.75.1630 23646211 PMC3641921

[40] Biks GA, Tariku A, Wassie MM, Derso T. Mother’s Infant and Young Child Feeding (IYCF) knowledge improved timely initiation of complementary feeding of children aged 6–24 months in the rural population of northwest Ethiopia. BMC research notes. 2018;11(1):1–7. doi: 10.1186/s13104-017-3088-5 30115114 PMC6097428

