# Supplementary material for: Infant and young child feeding practice among mothers of children age 6 to 23 months in Debrelibanos district, North Showa zone, Oromia region, Ethiopia
Source: PLoS One. 2021 Sep 24;16(9):e0257758. doi: 10.1371/journal.pone.0257758 (PMC8462673; doi:10.1371/journal.pone.0257758)
Supplement: S1 File — (DOCX) [file pone.0257758.s002.docx]

English Version Questionnaire

Identification: ________________
Health institutions: ________________________

Greetings:
Hello, my name is ______________. I am working in the research team of Salale University. I would like to interview you a few questions about factors affecting complementary feeding practice and associated factors. The objective of this study is to assess complementary feeding practice and associated factors among mothers of infants 6-23 months old visiting health institutions. Your cooperation and willingness for the interview is helpful in identifying problems related to the subject matter. Your name will not be written in this form. All information that you give will be kept strictly confidential. Your participation is voluntary and you are not obliged to answer any question you do not wish to answer. If you are still feeling discomfort with the interview please feel free to drop it any time you want.

Do I have your permission to continue?
1. Yes, ________________________, then continue to the next page.
2. No__________ asks the reason, thank the patient and skip to the next respondent.

Date of interview _______________ Time started___________ Time finished_________
Supervisor’s name ________________signature __________________

| **PART I: SOCIO-DEMOGRAPHIC CHARACTERISTICS OF MOTHER** | | | | |
| --- | --- | --- | --- | --- |
| Sr.N | Questions | Possible answer | Skip | |
| 101 | Age | _______________ |  | |
| 102 | Marital status | 1. Married 2. Single 3. Divorced 4. Widowed |  | |
| 103 | Religion | 1)Orthodox  3) Protestant  3) Muslim  4) Others­­_____________ |  | |
| 104 | Occupation of mother | 1. House wife 2. Government employee 3. Private employee 4. merchant 5. others (specify) |  | |
| 105 | Ethnicity | 1. Oromo 2. Amhara 3. Tigre 4. Other, specify____________ |  | |
| 106 | Educational status of mother | 1. Unable to read and write 2. able to read and write 3. primary school 4. secondary school 5. College and above |  | |
| 108 | husband’s educational status | 1) Unable to read and write  2) able to read and write  3) primary school  4) secondary school  5) College and above |  | |
| 109 | Occupation of husband | 1. Farmer  2. Merchant / trade  3. Private organization employee  4. Government employee  5. Daily laborer  6. Other (specify |  | |
| 110 | Family monthly income | ________ETB |  | |
|  | **PART II:MATERNAL AND HOUSEHOLD CHARACTERISTICS** | |  | |
| 201 | Parity of mother | ________________ |  | |
| 203 | Place of delivery | 1. Home 2. Public hospital 3. Health center 4. Private clinic |  | |
| 204 | ANC follow up | 1. Yes 2. No |  | |
| 205 | Attend PNC | 1. Yes 2. No |  | |
| 206 | Number of ANC visit | _________________ |  | |
| 207 | Preceding birth interval | ________________ |  | |
| 208 | Current pregnancy status | 1. Yes 2. No |  | |
| 209 | Complementary feeding information during EPI | 1. Yes 2. No |  | |
| 210 | Husband involvement in infant feeding | 1. Yes 2. No |  | |
| 211 | TV or Radio possession | 1. Yes 2. No |  | |
| 212 | Number of children excluding infant in the study | _____________ |  | |
|  | **PART III: CHILD CHARACTERISTICS** | |  | |
| 301 | Age of the child | _____________ |  | |
| 302 | Sex of the child | 1. Male 2. Female |  | |
| 303 | Birth order | ________________ |  | |
| 304 | Sickness episode within last 2 weeks | 1. Yes 2. No |  | |
| 305 | Perceived weight of child at birth | 1. Under weight 2. Normal 3. Overweight |  | |
| **PART V: KNOWLEDGE OF MOTHER/CAREGIVER ON IYC FEEDING** | | | | |
| 401 | Source of information on complementary feeding | 1. Television 2. Radio 3. Health professional 4. Housebound 5. Friend 6. Elder mother 7. Other |  | |
| 402 | Exposure to IYCF education | 1. Yes 2. No |  | |
| 403 | Water and herbal drinks should be given between 0-6 months | 1. Yes 2. No |  | |
| 404 | Breast feeding should continue till 24months | 1. Yes 2. No |  | |
| 405 | Complementary feeding should start at 6mon | 1. Yes 2. No |  | |
| 406 | Local foods should be used as Complementary foods | 1. Yes 2. No |  | |
| 407 | Eggs and other animal protein should be given from 6-24 months. | 1. Yes 2. No |  | |
| ***Part IV IYC FEEDING PRACTICE*** | | | | |
| 501 | Ever breast feed your child | 1. Yes 2. No | |  |
| 503 | Still breast feeding your child | 1. Yes 2. No | |  |
| 504 | Age at which child stop breast feeding | 1. Yes 2. No | |  |
| 505 | Ever started complementary feeding your child | 1. Yes 2. No | |  |
| 506 | When did you start complementary feeding | ________________ | |  |
| 507 | Did you use separate container to feed your child? | 1. Yes 2. No | |  |
| 508 | Type of separate container used? | 1. Bottle 2. Cup with spoon 3. Other (specify) | |  |
| 509 | Number of times you feed your child per day | ________________ | |  |
| 510 | Do you include snack between foods | 1. Yes 2. No | |  |
| 511 | Consistency of food used mostly? | 1. Porridge 2. Gruel 3. Adult type | |  |
| 512 | What food did you give your child within the past 24 hours? | 1. grain, tubers and tubers 2. lugems and nuts 3. diary product 4. Flesh foods 5. Egg 6. Vit A rich fruits and vegetables 7. Other fruits and vegetables | |  |
| PART VI: attitude of mother towards Complementary feeding | | | | |
| 601 | I provide complementary foods because it makes my infant fat | 1. Strongly agree 2. Agree 3. Neutral 4. Disagree 5. Strongly disagree | |  |
| 602 | I have enough money to buy complementary food items instead of suffering myself by breast feeding | 1. Strongly agree  2. Agree  3. Neutral  4. Disagree  5. Strongly disagree | |  |
| 603 | My breast milk is not sufficient to my infant so just after birth I like to introduce complementary foods to my infant | 1. Strongly agree  2. Agree  3. Neutral  4. Disagree  5. Strongly disagree | |  |
| 604 | Since others can help me by providing complementary foods I like it | 1. Strongly agree  2. Agree  3. Neutral  4. Disagree  5. Strongly disagree | |  |
| 605 | Breast feeding makes my appearance thin so I like to give complementary foods to my infant | 1. Strongly agree  2. Agree  3. Neutral  4. Disagree  5. Strongly disagree | |  |
| 607 | Providing my infant with complementary foods make him/her healthy and strong | 1. Strongly agree 2. Agree 3. Neutral 4. Disagree 5. Strongly disagree | |  |
| 608 | After six months complementary foods in addition of breast feeding are preferable. | 1. Strongly agree  2. Agree  3. Neutral  4. Disagree  5. Strongly disagree | |  |

Thank you for your cooperation!!!
