# Supplementary material for: Infant and young child feeding practice among mothers of children age 6 to 23 months in Debrelibanos district, North Showa zone, Oromia region, Ethiopia
Source: PLoS One. 2021 Sep 24;16(9):e0257758. doi: 10.1371/journal.pone.0257758 (PMC8462673; doi:10.1371/journal.pone.0257758)
Supplement: Attachment — Submitted filename: Response to Reviewers.docx [file pone.0257758.s003.docx]

**PONE-D-20-34804
Infant and Young Child Feeding Practice among Mothers with Children age between 6 to 23 Months at Debrelibanos District, North Showa, Oromia Ethiopia**
PLOS ONE
Dear editor,

Pleases find the revised manuscript. We have incorporated all comments recommended by the academic editor and reviewers. Following is the point-wise reply to the academic editor’s and reviewer’s comments.

**As per your suggestion, we have now submitted the following documents:**

- A rebuttal letter that responds to comments raised by the reviewers. This is being uploaded as a separate file and labelled **'Response to Reviewers'.**
- A marked-up copy of our manuscript that highlights changes made to the original version. This is being uploaded as a separate file and labelled '**Revised Manuscript with Track Changes'**.
- An unmarked version of the revised paper without tracked changes. This is being uploaded as a separate file and labelled **'Revised Manuscript'**.

With best regards,

Mr. Mathewos Mekonnen Gemmechu

**Journal Requirements:**

1. Please ensure that your manuscript meets PLOS ONE's style requirements, including those for file naming. The PLOS ONE style templates can be found at

https://journals.plos.org/plosone/s/file?id=wjVg/PLOSOne_formatting_sample_main_body.pdf and

[https://journals.plos.org/plosone/s/file?id=ba62/PLOSOne_formatting_sample_title_authors_affiliations.pdf](about:blank)

***Reply: Thank you for the guidance, the PLOS ONE’s style requirement have been incorporated accordingly in all sections.***

2. We suggest you thoroughly copyedit your manuscript for language usage, spelling, and grammar. If you do not know anyone who can help you do this, you may wish to consider employing a professional scientific editing service.  

Whilst you may use any professional scientific editing service of your choice, PLOS has partnered with both American Journal Experts (AJE) and Editage to provide discounted services to PLOS authors. Both organizations have experience helping authors meet PLOS guidelines and can provide language editing, translation, manuscript formatting, and figure formatting to ensure your manuscript meets our submission guidelines. To take advantage of our partnership with AJE, visit the AJE website ([http://learn.aje.com/plos/](about:blank)) for a 15% discount off AJE services. To take advantage of our partnership with Editage, visit the Editage website (www.editage.com) and enter referral code PLOSEDIT for a 15% discount off Editage services.  If the PLOS editorial team finds any language issues in text that either AJE or Editage has edited, the service provider will re-edit the text for free.

Upon resubmission, please provide the following:

- The name of the colleague or the details of the professional service that edited your manuscript
- A copy of your manuscript showing your changes by either highlighting them or using track changes (uploaded as a *supporting information* file)
- A clean copy of the edited manuscript (uploaded as the new *manuscript* file)

***Reply: Based on your advices*** ***the whole manuscript has been copy edited thoroughly for language usage, spelling and grammar by one of our colleagues not listed as author, who is expert in English.***

3. PLOS requires an ORCID iD for the corresponding author in Editorial Manager on papers submitted after December 6th, 2016. Please ensure that you have an ORCID iD and that it is validated in Editorial Manager. To do this, go to ‘Update my Information’ (in the upper left-hand corner of the main menu), and click on the Fetch/Validate link next to the ORCID field. This will take you to the ORCID site and allow you to create a new iD or authenticate a pre-existing iD in Editorial Manager. Please see the following video for instructions on linking an ORCID iD to your Editorial Manager account: https://www.youtube.com/watch?v=_xcclfuvtxQ

***Reply: The corresponding author has already ORCID iD (0000-0002-3794-7818)***

4. Please amend the manuscript submission data (via Edit Submission) to include author Tadele Kinati, Kumera Bekele, Bikila Tesfa, Dejene Hailu, and Kemal Jemal.

***Reply: The manuscript submission data has been amended accordingly***

5. Please include captions for your Supporting Information files at the end of your manuscript, and update any in-text citations to match accordingly. Please see our Supporting Information guidelines for more information: [http://journals.plos.org/plosone/s/supporting-information](about:blank).

***Reply: Modified accordingly***

**Additional Editor Comments:

Dear Authors**

The manuscript touches on an important topic Infant and Young Child Feeding (IYCF) in Ethopia. It investigates the prevalence of appropriate parctice and factors associated with appropriate practice to improve communication and practice in future.
While the paper presents interesting results and compares them with former research on the topic, the results are insufficiently presented and the comaprisons lack information on the former studies. Overall, the statistical methods can be presented better, to allow readers to interprete results better. The paper needs an English editing and a major revision.

***Reply: The result has been sufficiently presented and comparison on previous studies have been made thoroughly. The methodology has been presented in the better way so that it can be understood easily by reader. The language is copy edited***

Specific points:
Some sentences are not understandable and can be misinterpreted, e.g. "Mothers who were critically ill and not volunteer to participate in the study were excluded." Are these 2 exclusion criteria or one, mothers critically ill not volonteering....
In general non-participation is not an exclusion criterium.
***Reply: Modification has been made accordingly***
Authors should explain if the district chosen to study IYCF is representative of the country as a whole and if the results are generalizable.

***Reply: The issue of generatability has been discussed generously as limitation***
24 hour recall is mentioned among the operational definitions. It would be worthwhile to present the method in the data colelciton part and address how these data were analyzed.
***Reply: Modification has been made by authors accordingly***


Appropriate IYCF practices - is the threshold a standard threshold - no reference was cited. Please elaborate.
Similarly, please define "sufficient knowledge" when was it considered sufficient.
***Reply: Modifications have been made by authors accordingly***


Statistical methods: please add the confounding factors used in the multivariate regression analyses, or was this rather a explorative approach with all co-variates adjusting each other?

***Reply: The P value ≤0.25 was taken as a cut-off point to select eligible variables for the multiple logistic regression analysis to control for potential confounders*.**

Discussion: Authors present other studies on IYCF - however only vaguely refer to explanations for differences - such as might be due to socio-economic differences. This is not satisfying for the readers. Elaborate.
the two least well practiced recommendations (diversity in foods, bottle-feeding) merit more discussion, also due to the question of availability/access to diverse foods. In this case, education on IYCF success seem limeted.
The discussion should also include a section on generalizability.

***Reply: All comments raised in the discussion part have been addressed accordingly.***

Minor points:

Tbl. 1: familiy income - <1000 is twice

***Reply: Sorry for the incontinence, the second one has been removed.***

Gender bias, to me this does not seem to be a bias, but a gender difference in feeding practices. There is some literature on the topic and hypothesis on the reasons for this difference.

***Reply: authors have reviewed other literatures and modified accordingly.***

**Reviewers' comments:

Reviewer's Responses to Questions**

**Comments to the Author**

1. Is the manuscript technically sound, and do the data support the conclusions?

The manuscript must describe a technically sound piece of scientific research with data that supports the conclusions. Experiments must have been conducted rigorously, with appropriate controls, replication, and sample sizes. The conclusions must be drawn appropriately based on the data presented.

Reviewer #1: Partly

2. Has the statistical analysis been performed appropriately and rigorously?

Reviewer #1: No

***Reply: The procedure for selecting the candidate variables for the multivariate analysis has been stated.***

3. Have the authors made all data underlying the findings in their manuscript fully available?

The [PLOS Data policy](http://www.plosone.org/static/policies.action#sharing) requires authors to make all data underlying the findings described in their manuscript fully available without restriction, with rare exception (please refer to the Data Availability Statement in the manuscript PDF file). The data should be provided as part of the manuscript or its supporting information, or deposited to a public repository. For example, in addition to summary statistics, the data points behind means, medians and variance measures should be available. If there are restrictions on publicly sharing data—e.g. participant privacy or use of data from a third party—those must be specified.

Reviewer #1: No

***Reply: We have included the minimal data set in the manuscript as a separate attachment called ‘supporting information.***

4. Is the manuscript presented in an intelligible fashion and written in standard English?

PLOS ONE does not copyedit accepted manuscripts, so the language in submitted articles must be clear, correct, and unambiguous. Any typographical or grammatical errors should be corrected at revision, so please note any specific errors here.

Reviewer #1: No

***Reply: The whole manuscript has been copy edited thoroughly for language usage, spelling and grammar by colleague who is expert in English.***

5. Review Comments to the Author

Please use the space provided to explain your answers to the questions above. You may also include additional comments for the author, including concerns about dual publication, research ethics, or publication ethics. (Please upload your review as an attachment if it exceeds 20,000 characters)

Reviewer #1: I found that title of the manuscript was very interesting. However, the whole body of the manuscript was written in poor English. Even the language in the abstract seems a zero-draft document. The discussion is very shallow which leads to shallow conclusion. The binary and multivariate analysis should have been separately presented. Nothing was stated on how the candidate variables for the multivariate analysis were selected. What's the justification behind including all the 11 kebeles of the district? In general, this manuscript needs critical revision.

***Reply: Thank you for your constructive comments. The authors have taken all the raised issues and reacted accordingly.*** ***The whole manuscript has been copy edited thoroughly for language usage, spelling and grammar by colleague who is expert in English. Literature review has been made and discussed with the finding of this study in detail. Thus, sound conclusion has been drawn. The P value ≤0.25 was taken as a cut-off point to select eligible variables for the multiple logistic regression analysis to control for potential confounders Due to the small number of kebeles in the district, all of them were considered. In general, the manuscript has been critically revised by authors***

6. PLOS authors have the option to publish the peer review history of their article ([what does this mean?](https://journals.plos.org/plosone/s/editorial-and-peer-review-process#loc-peer-review-history)). If published, this will include your full peer review and any attached files.


If you choose “no”, your identity will remain anonymous but your review may still be made public.


**Do you want your identity to be public for this peer review?** For information about this choice, including consent withdrawal, please see our [Privacy Policy](https://www.plos.org/privacy-policy).

Reviewer #1: No

[NOTE: If reviewer comments were submitted as an attachment file, they will be attached to this email and accessible via the submission site. Please log into your account, locate the manuscript record, and check for the action link "View Attachments". If this link does not appear, there are no attachment files.]

While revising your submission, please upload your figure files to the Preflight Analysis and Conversion Engine (PACE) digital diagnostic tool, <https://pacev2.apexcovantage.com/>. PACE helps ensure that figures meet PLOS requirements. To use PACE, you must first register as a user. Registration is free. Then, login and navigate to the UPLOAD tab, where you will find detailed instructions on how to use the tool. If you encounter any issues or have any questions when using PACE, please email PLOS at [figures@plos.org](mailto:figures@plos.org). Please note that Supporting Information files do not need this step.

*In compliance with data protection regulations, you may request that we remove your personal registration details at any time.* [*(Remove my information/details)*](https://www.editorialmanager.com/pone/login.asp?a=r)*. Please contact the publication office if you have any questions.*
