# Supplementary material for: Infant and young child feeding practice among mothers of children age 6 to 23 months in Debrelibanos district, North Showa zone, Oromia region, Ethiopia
Source: PLoS One. 2021 Sep 24;16(9):e0257758. doi: 10.1371/journal.pone.0257758 (PMC8462673; doi:10.1371/journal.pone.0257758)
Supplement: Attachment — Submitted filename: Response to Reviewers.docx [file pone.0257758.s004.docx]

**PONE-D-20-34804
Infant and Young Child Feeding Practice among Mothers with Children age between 6 to 23 Months at Debrelibanos District, North Showa, Oromia Ethiopia**
PLOS ONE
Dear editor,

Pleases find the revised manuscript. We have incorporated all comments recommended by the academic editor and reviewers. Following is the point-wise reply to the academic editor’s and reviewer’s comments.

**As per your suggestion, we have now submitted the following documents:**

- A rebuttal letter that responds to comments raised by the reviewers. This is being uploaded as a separate file and labelled **'Response to Reviewers'.**
- A marked-up copy of our manuscript that highlights changes made to the original version. This is being uploaded as a separate file and labelled '**Revised Manuscript with Track Changes'**.
- An unmarked version of the revised paper without tracked changes. This is being uploaded as a separate file and labelled **'Revised Manuscript'**.

With best regards,

Mr. Mathewos Mekonnen Gemmechu

Journal Requirements:

Please review your reference list to ensure that it is complete and correct. If you have cited papers that have been retracted, please include the rationale for doing so in the manuscript text, or remove these references and replace them with relevant current references. Any changes to the reference list should be mentioned in the rebuttal letter that accompanies your revised manuscript. If you need to cite a retracted article, indicate the article’s retracted status in the References list and also include a citation and full reference for the retraction notice.

***Reply: Thank you for guidance. Authors have checked the reference list and ensured that it is complete and correct. Authors have also not cited papers that have been retracted.***

Additional Editor Comments (if provided):

Please address the last points raised by the reviewers.

***Reply: The points that had been raised by reviewers have been addressed accordingly.***


[Note: HTML markup is below. Please do not edit.]

Reviewers' comments:

Reviewer's Responses to Questions

**Comments to the Author**

1. If the authors have adequately addressed your comments raised in a previous round of review and you feel that this manuscript is now acceptable for publication, you may indicate that here to bypass the “Comments to the Author” section, enter your conflict of interest statement in the “Confidential to Editor” section, and submit your "Accept" recommendation.

Reviewer #1: (No Response)

Reviewer #2: (No Response)

2. Is the manuscript technically sound, and do the data support the conclusions?

The manuscript must describe a technically sound piece of scientific research with data that supports the conclusions. Experiments must have been conducted rigorously, with appropriate controls, replication, and sample sizes. The conclusions must be drawn appropriately based on the data presented.

Reviewer #1: Yes

Reviewer #2: Partly

3. Has the statistical analysis been performed appropriately and rigorously?

Reviewer #1: Yes

Reviewer #2: I Don't Know

4. Have the authors made all data underlying the findings in their manuscript fully available?

The [PLOS Data policy](http://www.plosone.org/static/policies.action#sharing) requires authors to make all data underlying the findings described in their manuscript fully available without restriction, with rare exception (please refer to the Data Availability Statement in the manuscript PDF file). The data should be provided as part of the manuscript or its supporting information, or deposited to a public repository. For example, in addition to summary statistics, the data points behind means, medians and variance measures should be available. If there are restrictions on publicly sharing data—e.g. participant privacy or use of data from a third party—those must be specified.

Reviewer #1: No

Reviewer #2: Yes

***Reply for Reviewer #1: We have included the additional data set in the manuscript as a separate attachment called ‘supporting information’ as S1 and S2***

5. Is the manuscript presented in an intelligible fashion and written in standard English?

PLOS ONE does not copyedit accepted manuscripts, so the language in submitted articles must be clear, correct, and unambiguous. Any typographical or grammatical errors should be corrected at revision, so please note any specific errors here.

Reviewer #1: Yes

Reviewer #2: No

***Reply for Reviewer #2: The whole manuscript has been copy edited thoroughly for language usage, spelling and grammar.***

6. Review Comments to the Author

Please use the space provided to explain your answers to the questions above. You may also include additional comments for the author, including concerns about dual publication, research ethics, or publication ethics. (Please upload your review as an attachment if it exceeds 20,000 characters)

Reviewer #1: It is appreciated that almost all the raised issues are now addressed except the following few concerns.
1. The authors have also responded that the minimal dataset is separately attached as ‘supporting information. However the dataset underlying the findings in the manuscript is still not accessible if I am not mistaken.

2. Some statements still need minor editions. For example, something is wrong in the statement written as, "In this study, mothers who made ANC follow up during pregnancy were statistically significant with appropriate IYCF practices."
I think this should be rephrased in a way that the information is clearly presented.

Reviewer #2: The manuscript is about IYCF practices and their associated factors.

In general the manuscript needs extensive language and grammar editing. In the introduction section, why isn't 2016 EDHS used to give some background ? In the results section, table 4 (binary and multivariate regression analysis) is not easy to read/understand, it would be better to present results in two tables and use no more than one page per table. In the discussion section, the binary and multivariate analysis results need to be discussed with more depth.

***Reply for Reviewer #1: Thank you for your constructive comments. We have included the additional data set in the manuscript as a separate attachment called ‘supporting information’ i.e. S2. Authors have also edited statements that need editions so that it can be clearly understood by readers clearly.***

***Replay for Reviewer #2: Thank you for taking the time to give your valuable feedback. To address the concerns identified, the entire manuscript has been thoroughly edited for language usage, spelling, and grammar. Secondly, one paragraph in the introductory section has been dedicated to providing some background information utilizing the 2016 EDHS. Thirdly, the authors improved the design and format of Table 4 in the results section to make it more understandable.Finally, the authors have added depth the discussion of multivariate analysis in the discussion section. However, related to binary analysis, it was already adjusted after considering all variables with P-value ≤0.25 in binary analysis for the multivariate logistic regression analysis to control for potential confounders.***

***Thank you once again for your constructive comments!***

7. PLOS authors have the option to publish the peer review history of their article ([what does this mean?](https://journals.plos.org/plosone/s/editorial-and-peer-review-process#loc-peer-review-history)). If published, this will include your full peer review and any attached files.


If you choose “no”, your identity will remain anonymous but your review may still be made public.


**Do you want your identity to be public for this peer review?** For information about this choice, including consent withdrawal, please see our [Privacy Policy](https://www.plos.org/privacy-policy).

Reviewer #1: No

Reviewer #2: No

[NOTE: If reviewer comments were submitted as an attachment file, they will be attached to this email and accessible via the submission site. Please log into your account, locate the manuscript record, and check for the action link "View Attachments". If this link does not appear, there are no attachment files.]

While revising your submission, please upload your figure files to the Preflight Analysis and Conversion Engine (PACE) digital diagnostic tool, <https://pacev2.apexcovantage.com/>. PACE helps ensure that figures meet PLOS requirements. To use PACE, you must first register as a user. Registration is free. Then, login and navigate to the UPLOAD tab, where you will find detailed instructions on how to use the tool. If you encounter any issues or have any questions when using PACE, please email PLOS at [figures@plos.org](mailto:figures@plos.org). Please note that Supporting Information files do not need this step.
