# Supplementary material for: Infant and young child feeding practice among mothers of children age 6 to 23 months in Debrelibanos district, North Showa zone, Oromia region, Ethiopia
Source: PLoS One. 2021 Sep 24;16(9):e0257758. doi: 10.1371/journal.pone.0257758 (PMC8462673; doi:10.1371/journal.pone.0257758)
Supplement: Attachment — Submitted filename: Response to Reviewers.docx [file pone.0257758.s005.docx]

**Infant and Young Child Feeding Practice among Mothers with Children age between 6 to 23 Months at Debrelibanos District, North Showa, Oromia Ethiopia**
PLOS ONE
Dear editor,

Pleases find the revised manuscript. We have incorporated all comments recommended by the academic editor and journal requirements. Following is the point-wise reply to the academic editor’s comments and journal requirements.

**As per your suggestion, we have now submitted the following documents:**

- A rebuttal letter that responds to comments raised by the reviewers. This is being uploaded as a separate file and labelled **'Response to Reviewers'.**
- A marked-up copy of our manuscript that highlights changes made to the original version. This is being uploaded as a separate file and labelled '**Revised Manuscript with Track Changes'**.
- An unmarked version of the revised paper without tracked changes. This is being uploaded as a separate file and labelled **'Revised Manuscript'**.

Journal Requirements:

Please review your reference list to ensure that it is complete and correct. If you have cited papers that have been retracted, please include the rationale for doing so in the manuscript text, or remove these references and replace them with relevant current references. Any changes to the reference list should be mentioned in the rebuttal letter that accompanies your revised manuscript. If you need to cite a retracted article, indicate the article’s retracted status in the References list and also include a citation and full reference for the retraction notice.

***Replay: Up on reviewing the reference list and the whole citations in the manuscript, the following modifications have been made. The references are now ensured to be relevant, up-to-date, complete and correct. Thank you for your guidance.***

- ***Reference N^O^ -3 (Pollitt E, Gorman KS, Engle PL, Martorell R, Rivera J, Wachs TD, et al. Early supplementary feeding and cognition: effects over two decades. Monographs of the Society for Research in Child Development. 1993:i-118) is replaced by ( Opoola F, Adebisi SS, Ibegbu AO. The Study of nutritional status and academic performance of primary school children in zaria, Kaduna state, Nigeria. Annal Bioanthropol. 2016;4(2):96)*** ***which is more relevant and up-to-date.***
- ***Reference N^O^ -8 (Daelmans B, Dewey K, Arimond M. New and updated indicators for assessing infant and young child feeding. Food and nutrition bulletin. 2009; 30(2_suppl2) :256-62.) is replaced by reference ( Benson T, Bellete S, Chanyalew D, Belachew T. An assessment of the causes of malnutrition in Ethiopia. International Food Policy Research Institute Washington DC. 2005; 1-213.) which is more up-to-date and more relevant.***
- ***In paragraph 2 of Introduction Section references cited as [2-4] are now corrected as reference citation as [10 -12]***
- ***Reference N^O^ - 13, 16 & 33 are modified into correct Vancouver referencing style***
- ***In Discussion section statement are properly cited by reference in the list where it is deemed (Please see Paragraph 7, 8 & 12)***

Additional Editor Comments (if provided):

The paper has largely improved. Thank you for providing another revision.

Please address the following points:
1. There seems to be a misunderstanding with regard to "data availability" - the authors added supplemental material S1 and S2 which are actually questionnaires/ questionnaire items used in the study. Data availability refers to the data being available on a data server or such. Please address, if it is possible or foreseen to publish the data once the paper is published. If not, please explain. Please see: https://journals.plos.org/plosone/s/data-availability

***Reply: Thank you for your constructive comment. Authors have now added Data Set used in the study as “S1 Data (SAV)” in addition to questionaries used in the study which has been submitted as “S 1 File (DOCX)” under title “Supporting information” right after Conclusion in manuscript. All relevant data are declared to be within the manuscript and its Supporting Information files during submission process.***

2. The first paragraph of the discussion is mainly a repetition of results already found in the result section even with percentages. Please rewrite the first paragraph summing up the main results respectively the main insights, without this detail. You can of course repeat results in the discussion, when you put them into comparison with literature or when interpreting them.

***Reply: Modification has been made accordingly.***

3. Table 4: please separate COR/AOR values from the CI values: 12.859(6.135,26.955) => 12.859 (6.135,26.955) and provide the same number of digits after the comma for all values. Add definition of COR and AOR to the legend.

***Reply: Modification has been made accordingly.***

[Note: HTML markup is below. Please do not edit.]

Reviewers' comments:

[NOTE: If reviewer comments were submitted as an attachment file, they will be attached to this email and accessible via the submission site. Please log into your account, locate the manuscript record, and check for the action link "View Attachments". If this link does not appear, there are no attachment files.]

While revising your submission, please upload your figure files to the Preflight Analysis and Conversion Engine (PACE) digital diagnostic tool, <https://pacev2.apexcovantage.com/>. PACE helps ensure that figures meet PLOS requirements. To use PACE, you must first register as a user. Registration is free. Then, login and navigate to the UPLOAD tab, where you will find detailed instructions on how to use the tool. If you encounter any issues or have any questions when using PACE, please email PLOS at [figures@plos.org](mailto:figures@plos.org). Please note that Supporting Information files do not need this step.

*In compliance with data protection regulations, you may request that we remove your personal registration details at any time.* [*(Remove my information/details)*](https://www.editorialmanager.com/pone/login.asp?a=r)*. Please contact the publication office if you have any questions.*
